# Socket shield technique for immediate implant placement after dental trauma: a case report

**DOI:** 10.1093/jscr/rjaf277

**Published:** 2025-05-07

**Authors:** Hai-yang Tang, Xin Li, Hong-jie Song

**Affiliations:** Department of Stomatology, Chengdu Second People’s Hospital, Sichuan University, No. 10, Qingyun South Street, Jinjiang Zone, Chengdu 610021, Sichuan Province, China; North Sichuan Medical College, No. 55 Dongshun Road, Gao Ping District, Nanchong, 637000, Sichuan Province, China; Department of Stomatology, Chengdu Second People’s Hospital, Sichuan University, No. 10, Qingyun South Street, Jinjiang Zone, Chengdu 610021, Sichuan Province, China; North Sichuan Medical College, No. 55 Dongshun Road, Gao Ping District, Nanchong, 637000, Sichuan Province, China

**Keywords:** dental trauma, esthetic zone, immediate restoration, immediate implantation, socket shield technique, case report

## Abstract

Anterior teeth are often extracted due to dental trauma, periodontal disease, and other factors. During the healing period of sockets, the loose bundle bone in the buccal side of the anterior teeth usually produces vertical and horizontal bone absorption. How to remain and reconstruct alveolar crest contour in the esthetic zone is a challenge to dentists. In this case, a 26-year-old man reported with the chief complaint of a traumatic fracture in upper front teeth region, accompanied by pain. After examination, he was diagnosed with tooth fracture (11,12 crown-root fracture). The socket shield technique was planned for treatment of tooth 11. Meanwhile, we converted the broken tooth as a temporary crown. After 6 months healing period, a porcelain crown was placed on the custom abutment. Through a 1-year follow-up, the alveolar crest contour was still stable. Consequently, socket shield technique can effectively maintain alveolar crest contour.

## Introduction

Various methods for alveolar ridge preservation, such as immediate implantation, soft or hard tissue augmentation, and non-flap implantation, have been extensively performed in implant clinics with the aim of coping with absorption and collapse of labial bone following tooth extraction [1]. Immediate implantation, which has a similar clinical success rate to that of conventional delayed implantation, can shorten the treatment course and alleviate postoperative pain [2]. However, immediate implantation is often accompanied by varying degrees of soft and hard tissue changes around the implant [3]. In the past few years, the socket shield technique has been applied as an alternative technique to immediate implantation [4]. Notably, the technique selectively reserves the buccal segment of the root and periodontal membrane in immediate implant surgery, thereby avoiding buccal bone loss [5]. Results from animal studies have shown that the socket shield technique guarantees good osseointegration between the implant and the root plate [6]. Based on this, we report an application of the socket shield technique for successful treatment of a case of traumatic maxillary central incisor.

## Case presentation

### Chief complaints

A 26-year-old man reported with the chief complaint of a traumatic fracture in upper front teeth region, accompanied by pain.

### History of present illness

**Figure 1:**
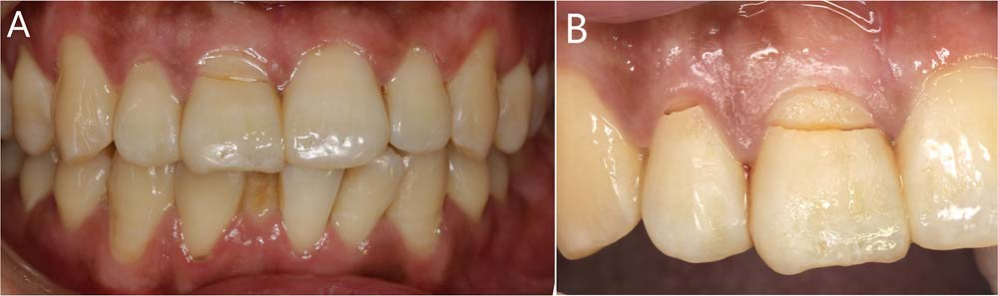
(A) The whole teeth photograph; (B) The fracture lines in teeth 11 and 12.

The patient fell down the stairs and injured his maxillary anterior teeth one day before (Fig. 1).

### Intraoral examination

Midline smile line; gingivitis with bleeding on probing; absence of occlusal interference. Severe percussion pain (+++) was observed in teeth 11 and 12. Teeth 11 and 12 exhibited crown fracture with grade-III mobility of the fractured segment.

### Imaging examinations

**Figure 2:**
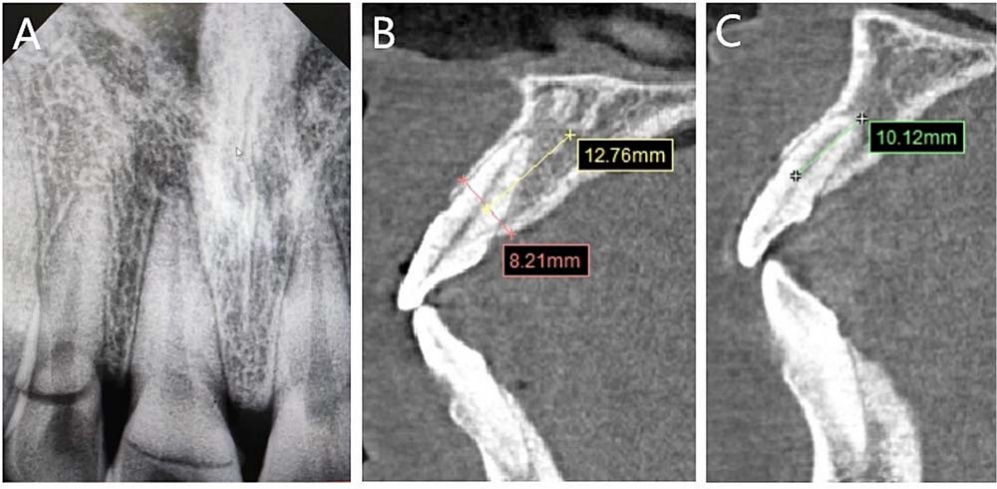
Imaging examinations. (A) The dental film; (B) 11 tooth position in CBCT images; (C) 12 tooth position in CBCT images.

Preoperative dental film revealed 11 and 12 crown-root fracture (Fig. 2A), while results from cone beam computed tomography (CBCT) showed that the lingual fracture line of 11 tooth was located under the alveolar bone, the buccal bone was thin (<1 mm), and the height of alveolar bone was sufficient (Fig. 2B). The lingual side of 12 tooth crown was located at the top of the alveolar crest, with a root length of about 10 mm. No obvious periapical shadow was found (Fig. 2C).

### Final diagnosis

Crown-root fracture with pulp exposure (11, 12).

### Treatment

After communication with the patient, the socket shield technique was planned for treatment of the 11 tooth.

#### (i) Surgical implantation

**Figure 3:**
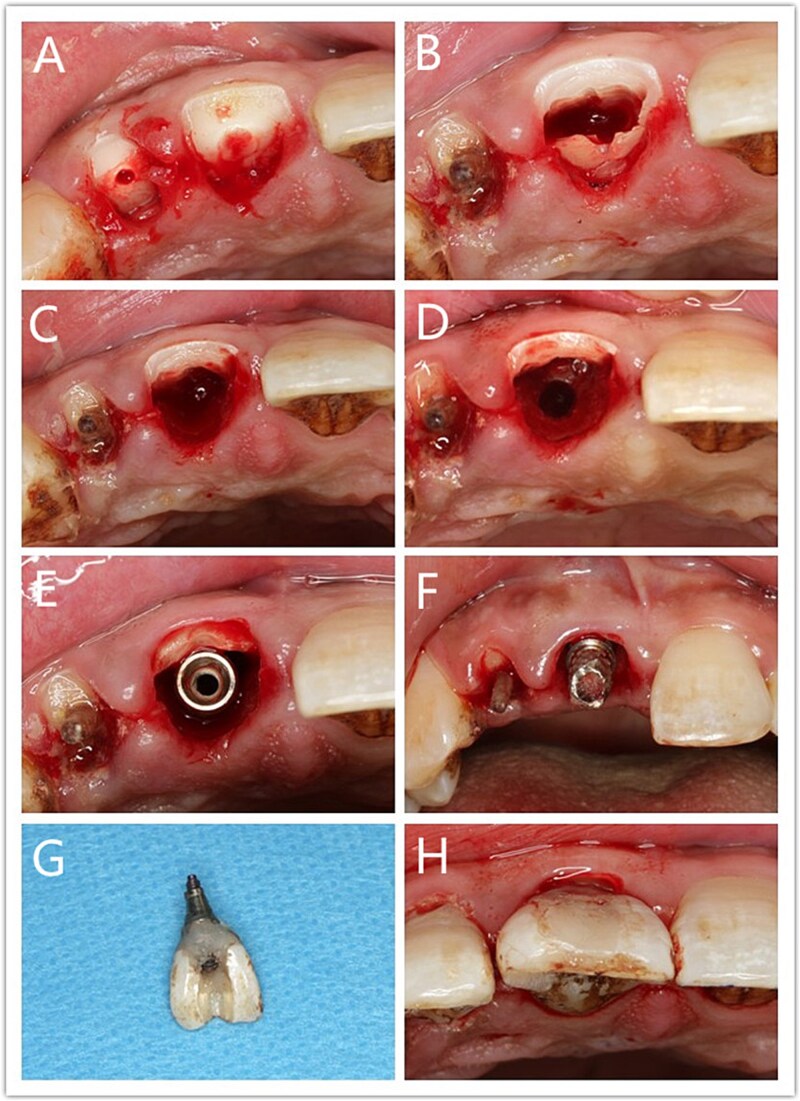
Socket shield technique and immediate restoration procedure; (A) Remove broken tooth tissues; (B) Separate the tooth root; (C) Remove the lingual root segment; (D) Prepare implant site; (E) Place implant; (F) Temporary abutment; (G) Temporary crown; (H) Temporary prosthesis.

The patient was given preoperative oral antibiotics, 0.5 h prior to the operation, with a 3-min gargling of compound chlorhexidine gargle. Local infiltration anesthesia was performed in the 11 tooth position after local disinfection. Once the effect of anesthesia was confirmed, a gingiva separator was used to separate the gingiva and remove broken tooth tissues at the crown of 11 tooth (Fig. 3A). Next, a turbine was used to separate the tooth root along the proximal and distal directions (Fig. 3B), removes the lingual root segment, and resizes the thickness of the buccal root segment (0.5–1.0 mm) (Fig. 3C). The socket was then gently scratched, with care taken to ensure integrity of the buccal root and no damage to the periodontal ligament during scratching. Thereafter, a hole was made at the lingual part of the extraction socket (Fig. 3D), under the guidance of a digital guide plate, and an implant (3.3×13 mm, Ezplus, Megagen, Korea) installed with an implant torque >35 N. cm [7], with a jumping space of about 1 mm [8] between implant and the residual root segment (Fig. 3E). After screwing it into the temporary abutment (Fig. 3F), a temporary crown was made by the fractured tooth crown (Fig. 3G). Using the natural crown as the temporary crown can reduce irritation to gums. The screw hole was finally sealed using resin (Fig. 3H).

#### (ii) Restoration treatment

**Figure 4:**
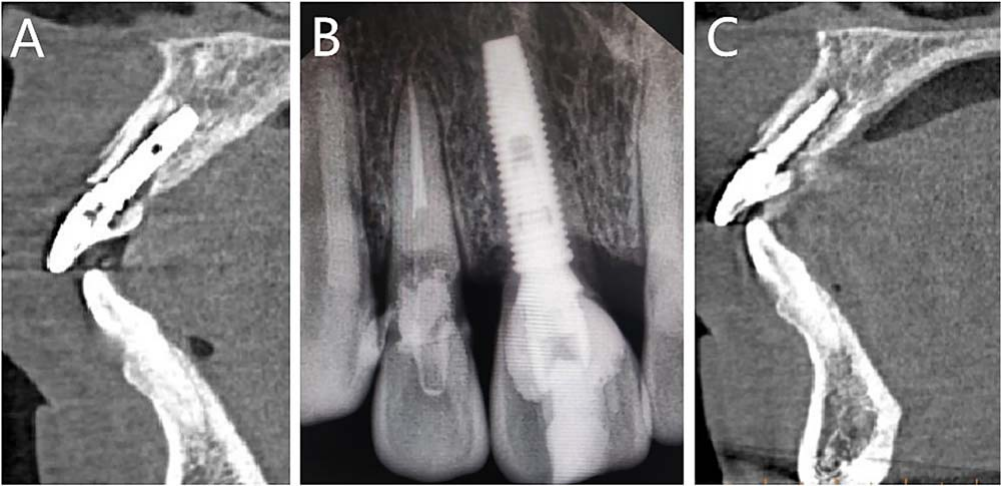
Follow-up imaging examinations. (A) Postoperative CT; (B) X-ray performed one month later; (C) CBCT performed 6 months later.

**Figure 5:**
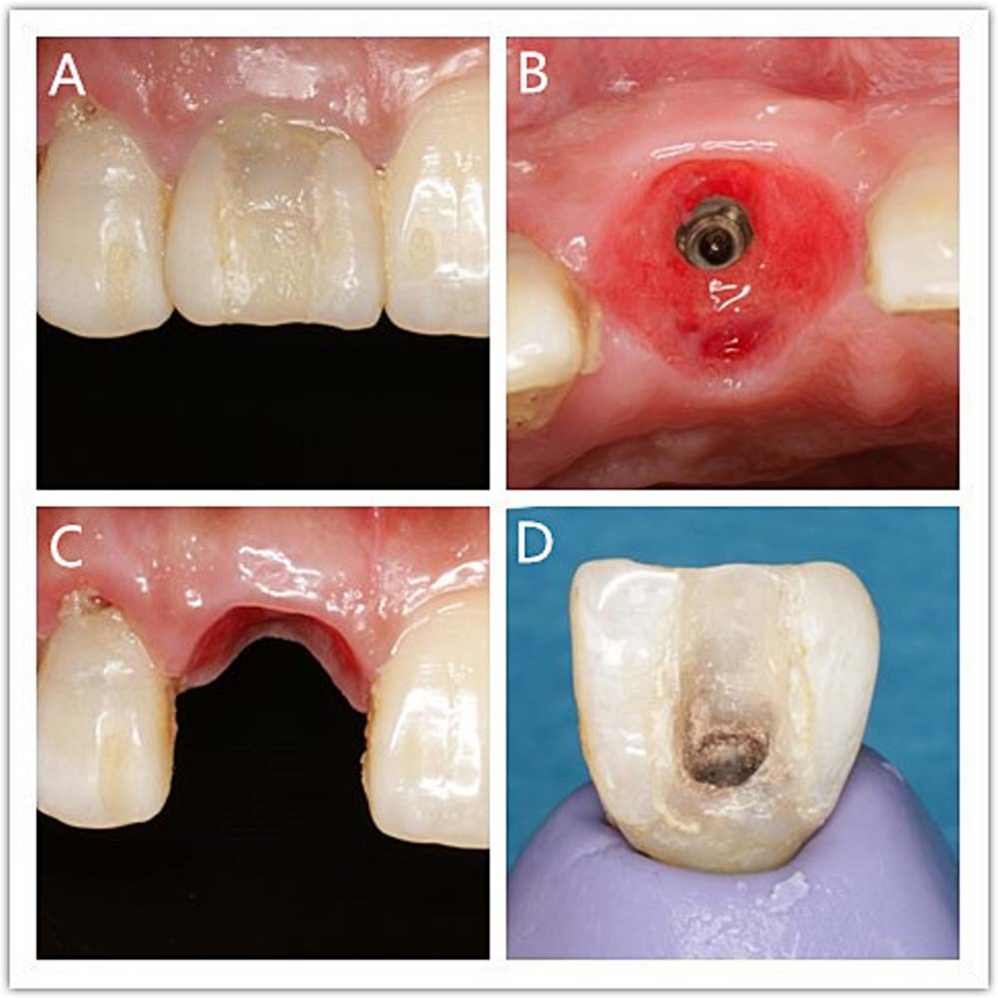
Personalized impression procedure. (A) The buccal gingival margin; (B) Emergence profile; (C) Gingival contour; (D) Personalized mold tool.

Postoperative CT scans revealed an intact buccal root segment, with a 1 mm distance between the implant and the segment (Fig. 4A). X-ray, performed one month later, showed that the implant was located in the middle of the edentulous area, while bone height was comparatively at the same level as the neck of the adjacent tooth in mesial and distal (Fig. 4B). CBCT, performed 6 months later, showed that the interval between the root segment and implant was filled with new bone tissue (Fig. 4C). The gingiva was in good condition, meanwhile, the soft tissue of the buccal side proliferated toward the incisal edge (Fig. 5A). Impression was performed with personalized mold tool to obtain individual emergence profile (Fig. 5B–D). Eventually, a porcelain crown was placed on the custom abutment.

### Outcome and follow-up

**Figure 6:**
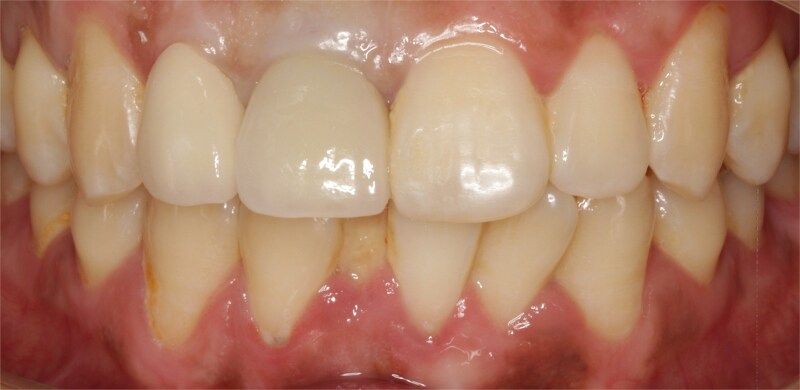
Final prosthesis.

The alveolar crest contour was still stable at 1 year of follow-up, and the patient was satisfied with his final prosthesis, which was in harmony with adjacent teeth (Fig. 6).

## Discussion

The socket shield technique, which requires no excessive surgical intervention, no application of bone graft materials, and no complex bone graft surgery, can effectively alleviate operative trauma, provide conditions for hard and soft tissue regeneration, and simultaneously reduce patient costs [9]. In the present case, we applied the socket shield technique to successfully maintain original alveolar crest contour. The unmoved buccal root segment guaranteed sufficient blood supply into the periodontal tissue. Meanwhile, we converted the broken tooth into a temporary crown. The converted tooth showed fewer stimuli to gum and more fitness to emergence profile rather than resin crown. Although only a handful of literature is currently available on the subject, the socket shield technique has shown promise in generating excellent effects on less soft and hard tissue absorption and conferring better results in the esthetic zone of anterior teeth than traditional implantation approaches [10]. At present, socket shield technique is still in the exploratory stage, and this technique remains associated with significant limitations [11–13]. First, the technique necessitates precise resection of the root apex while preserving a labial root fragment, a procedure that demands advanced surgical proficiency and meticulous control to avoid iatrogenic damage to surrounding tissues. However, clinical evidence indicates that failure risks are significantly associated with complications such as root exposure, progressive root resorption, and marginal bone loss around the implant [14]. Second, critical technical parameters—including the position, thickness, and height of the root fragment [15] and preparation strategies for roots with varying morphologies remain poorly defined, requiring systematic investigation to establish standardized protocols [16]. Third, existing studies addressing this technique are limited by small sample sizes, short follow-up periods (<24 months in most cases), and inconclusive data on long-term osseointegration outcomes [11, 17]. Therefore, high-quality randomized case–control studies, with large sample sizes, are required to provide a firm basis for application of this technique in teeth restoration.
